# Specific Ion Effects in Cholesterol Monolayers

**DOI:** 10.3390/ma9050340

**Published:** 2016-05-05

**Authors:** Teresa Del Castillo-Santaella, Julia Maldonado-Valderrama, Jordi Faraudo, Alberto Martín-Molina

**Affiliations:** 1 Departamento de Física Aplicada, Universidad de Granada, Campus de Fuentenueva sn, Granada 18071, Spain; tdelcastillo@ugr.es (T.D.C.-S.); julia@ugr.es (J.M.-V.); 2 Institut de Ciència de Materials de Barcelona (ICMAB-CSIC), Campus de la UAB, Bellaterra 08193, Spain

**Keywords:** ionic specificity, cholesterol, Langmuir monolayers, molecular dynamics simulations, surface forces

## Abstract

The interaction of ions with interfaces and, in particular, the high specificity of these interactions to the particular ions considered, are central questions in the field of surface forces. Here we study the effect of different salts (NaI, NaCl, CaCl_2_ and MgCl_2_) on monolayers made of cholesterol molecules, both experimentally (surface area *vs.* lateral pressure isotherms measured by a Langmuir Film Balance) and theoretically (molecular dynamics (MD) all-atomic simulations). We found that surface isotherms depend, both quantitatively and qualitatively, on the nature of the ions by altering the shape and features of the isotherm. In line with the experiments, MD simulations show clear evidences of specific ionic effects and also provide molecular level details on ion specific interactions with cholesterol. More importantly, MD simulations show that the interaction of a particular ion with the surface depends strongly on its counterion, a feature ignored so far in most theories of specific ionic effects in surface forces.

## 1. Introduction

The effect of ions is of key importance in determining physicochemical properties and functionality of systems as diverse as macromolecules, colloids, membranes, or microfluidic devices [1]. In spite of the substantial progress achieved in recent years in experimental, theoretical and simulations methods, several puzzling effects still lack a deep physical understanding. A particularly relevant and intriguing problem is the origin of ionic specificity [2]. By ionic specificity we mean that in a wide range of phenomena (surface forces, surface tensions, colloidal stability, protein stability, *etc*.) one finds that different ions, which have the same electrical charge, induce different behavior. These effects are particularly important in surface forces acting in biological systems [3]. These effects are important not only for monovalent ions but also for biologically relevant divalent ions such as Mg^2+^ or Ca^2+^ [4] for which electrostatics are usually assumed to be dominant.

In this work, we study the presence of specific ion effects in cholesterol monolayers at the air-water interface with different electrolytes. Cholesterol (CHOL) is an important constituent in cell membranes of most vertebrates being a major component of the plasma membrane of Eukaryotic cells [5]. CHOL affects cellular processes by interacting with other membrane lipids and specific proteins, and also participates in several membrane trafficking and transmembrane signaling processes [6,7]. Outside the field of fundamental biology, CHOL is an important element in the development of new biomaterials; as a helper lipid in liposomes developed for drug delivery [8,9] or in lipoplexes for gene therapy [10,11]. It is also an integral ingredient of new surfactant-based highly stable vesicles [12].

Given its influence on the structure and function of cell membranes, CHOL has been extensively studied since the late 18th century until present [6,7,13,14,15,16,17,18]. CHOL is a peculiar lipid, with a polar headgroup consisting only of one hydroxyl (OH) group (which is only weakly hydrophilic) and a strong hydrophobic region containing four hydrocarbon rings. Due to this chemical structure, it does not form bilayer structures. Instead it interacts strongly with other lipids, inducing a non-ideal behavior in mixed systems [13,14,19]. This non-ideal behavior, also referred as condensing effect, was first shown by Leathes in 1925, who demonstrated experimentally that the average area per molecule in mixtures of CHOL and egg lecithin monolayers was much lower than what would be expected from the individual components [20]. Since then, the condensing effect and the ability of sterols to induce ordering within the hydrocarbon chain has been extensively characterized using Langmuir films [17,19,21,22,23,24,25,26]. It is now clear that the presence of CHOL increases the ordering (cohesion and packing) of neighboring lipids [7] but, at the same time, contributes to increase the fluidity of the bilayer (fast lateral diffusion) [15]. Several theoretical schemes have been proposed to explain the mechanisms of CHOL-lipid interactions: the condensed-complexes model, the superlattice model, and the umbrella model (see [7,13,17] and references cited therein). In these models, the condensing effect is directly linked to the peculiar molecular structure of CHOL. In fact, experiments and simulations [13] have shown that no condensation is observed if the structure of the CHOL molecule is slightly modified by adding an additional hydrophilic head group, or if the size of the hydrophobic part of CHOL is reduced. In this sense, the study of CHOL monolayers may be of interest in systems in which the hydroxyl group of CHOL is functionalized, as has been proposed for biomedical applications [27].

Despite the ubiquitous presence of ions in biological systems, it is important to recall that the effect of ions on CHOL and on CHOL-lipid interactions has been studied only in a few works. However, there is increasing evidence of the impact of ions on systems containing cholesterol. For example, Korchowiec and co-workers have shown that the presence of cations (Na^+^ and Ca^2+^) increased monolayer condensation, stability and packing density in mixed 1,2-dipalmitoyl-sn-glycero-3-phosphoethanolamine (DPPE) and CHOL mixed films [26]. Condensation was more pronounced with Ca^2+^ as compared to Na^+^ ions. Another example is the case of ternary mixed monolayers containing CHOL and saturated phosphatidylethanolamine (PE) and phosphatidylserine (PS) in the presence of sodium or calcium ions [22]. In this case, the interactions between lipids are enhanced in the presence of Na^+^. Thus, it is clear that CHOL-lipid interactions are modulated by the presence of cations in the subphase. There are also a few studies that in addition to mixed systems, also consider pure CHOL monolayers. For example, in [26] the authors report isotherms of pure CHOL in the presence of three different subphases (pure water, 0.1 M of NaCl and 0.033 M of CaCl_2_). From these results, it can be seen that NaCl or CaCl_2_ have different effects on the isotherm. These specific ionic effects on CHOL monolayers deserve further study, since the underlying microscopic mechanism remains unknown as well as the effect of other ions.

Accordingly, the aim of this work is to carry out a systematic study of pure CHOL monolayers considering different combinations of cations and anions (NaCl, NaI, CaCl_2_ and MgCl_2_), hence extending previous experimental work also providing atomistic insights on these specific ionic effects. We use the Langmuir Film Balance as experimental technique (as in [26]) and molecular dynamics (MD) all-atomic simulations. We show that specific ion effects in cholesterol films show atypical and interesting features, which challenge our current understanding of specific ionic effects in surface forces.

## 2. Results

### 2.1. Interaction of Cholesterol Monolayers with NaCl

**Figure 1:**
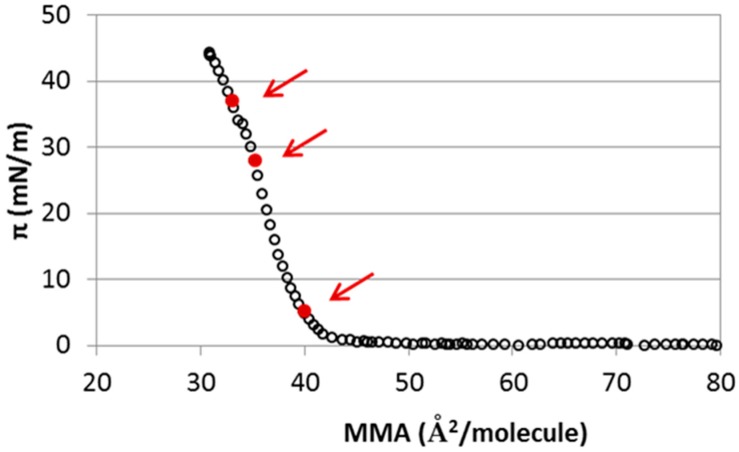
Surface pressure (π) *vs.* area per molecule (MMA) isotherm of cholesterol (CHOL) monolayers in a 15 mM NaCl subphase. The arrows indicate the particular cases analyzed by molecular dynamics (MD) simulations: 33.1 Å^2^/molecule; 35.3 Å^2^/molecule and; 40 Å^2^/molecule (standard deviation <2%).

Before discussing the results for different ions, we will first discuss the case of NaCl experimentally and using MD simulations. This case has been analyzed experimentally in the literature [26]. Figure 1 shows the surface pressure-area (π-A) isotherm obtained for a pure cholesterol monolayer on a subphase containing 15 mM NaCl at the air-water interface. The obtained experimental isotherm is consistent with previous experimental results shown in [24], and it is also similar to that obtained in pure water (see Figure 1 in [26]). The shape of the isotherm indicates that CHOL molecules remain in a 2D gaseous state upon compression of the monolayer to sharply enter into a condensed/solid phase as the compression proceeds. The steep rise in surface pressure is observed at 40 Å^2^/molecule, in close agreement with literature results [19,22,23,24,26,28]. This is known as the lift-off area. It corresponds to the transition from gaseous to solid state, with the CHOL molecules oriented with the hydroxyl group pointing towards the water phase and a condensed packing of the hydrophobic body of the CHOL molecule (see MD simulation results below). Another feature of interest of the isotherm is the collapse pressure, which is the highest pressure to which a monolayer can be compressed without a detectable movement of the molecules in the film to form a new phase. For CHOL monolayers, collapse occurs at 45 mN/m (Figure 1). The presence of 15 mM NaCl in the subphase does not change substantially the lift-off area or collapse pressure as compared with the results obtained in [22,26] for a CHOL monolayer in pure water. However, our results show slight differences in the shape of the isotherm in the presence of 15 mM NaCl, which appears slightly sharper, as compared with that obtained in pure water. This effect has been also observed for pure cholesterol monolayers in the presence of 100 mM NaCl [26]. This result can be interpreted by stating that there is an increased condensation effect at high surface pressures in the presence of NaCl [26].

**Figure 2:**
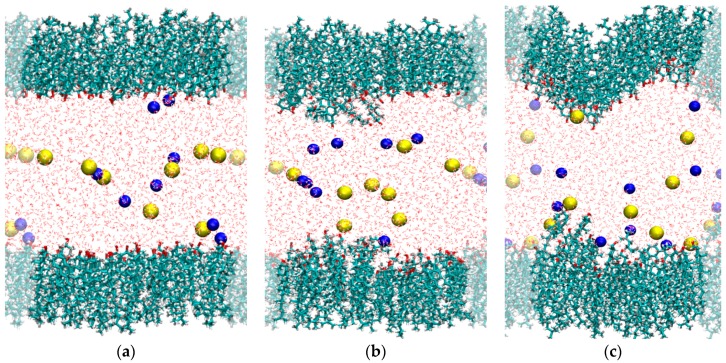
Snapshots from MD simulations (*T* = 298 K) corresponding to different area per molecule: (**a**) 40 Å^2^/molecule; (**b**) 35.3 Å^2^/molecule and; (**c**) 33.1 Å^2^/molecule. CHOL molecules are shown in bond representation; water is shown as lines and ions as spheres with Van der waals radii (yellow spheres: Na^+^; blue spheres: Cl^−^). The shaded region indicates the employed periodic boundary conditions. This figure was made with VMD software [29].

The π-A isotherms of spread monolayers (Figure 1) are macroscopic in nature, although the features of the isotherm are assumed to correspond to particular structural arrangements of the adsorbed monolayer. In order to provide an atomistic interpretation of the interaction of CHOL with NaCl, we have performed molecular dynamics simulations of CHOL monolayers at the air-water interface with full atomistic detail. The simulations were performed by considering a water slab in vacuum (air is not simulated) covered with a CHOL monolayer at each water interface, as seen in Figure 2 (see Section 4 for technical details). For the case of the NaCl subphase, we have considered three representative values of the area per molecule. First we considered 40 Å^2^/molecule (which is the lift-off area of the monolayer), and we also considered two different compression states of the monolayer: 35.3 Å^2^/molecule and; 33.1 Å^2^/molecule (these three values are marked in the isotherm, see Figure 1). As seen in the snapshots of Figure 2a, at the lift off value of the area per molecule (40 Å^2^/molecule) the monolayers are flat, with a very compact (condensed) packing of the cholesterol molecules. The –OH headgroup points towards the water phase and the hydrophobic core of the CHOL molecule is oriented perpendicular to the interface, maximizing close contacts between the planar rings of neighboring CHOL molecules. This molecular picture of the monolayer at 40 Å^2^/molecule supports the interpretation of the lift off area found in Figure 1 as a situation corresponding to a compact, condensed state. At this point, it is illustrative to think about what will happen at the molecular scale upon compression of this state of the monolayer. It seems clear that it is not possible to significantly reduce the separation between CHOL molecules which are already in close contact. Simulations performed at 35.3 Å^2^/molecule (Figure 2b) and 33.1 Å^2^/molecule (Figure 2c) provide a clue. When the monolayer is compressed at 35.3 Å^2^/molecule, there is a tendency of CHOL molecules to develop molecular-scale protrusions (Figure 2b) in order to obtain the space needed to allocate all the molecules of the monolayer (since CHOL molecules are insoluble). When the monolayer is further compressed at 33.1 Å^2^/molecule, protrusions of molecular size are not enough to provide the needed additional space and the monolayers develop local curvature, as seen in Figure 2c. It has to be emphasized that the curvature observed in Figure 2c is small (roughly between 1 and 2 nm), with a size on the order of the CHOL molecule. It is large enough to provide substantial additional space for CHOL molecules but small enough to be experimentally unobservable. Still, the development of such deformations requires substantial external pressure, which correlates to the steep rise in surface pressure observed in Figure 1 as the area per molecule in the monolayer decreases below 40 Å^2^/molecule. We also tried simulations at larger compressions, but it was not possible to obtain stable systems as the monolayer was close to collapse, in agreement with the experimentally observed surface pressures in Figure 1 at these compressions states.

**Figure 3:**
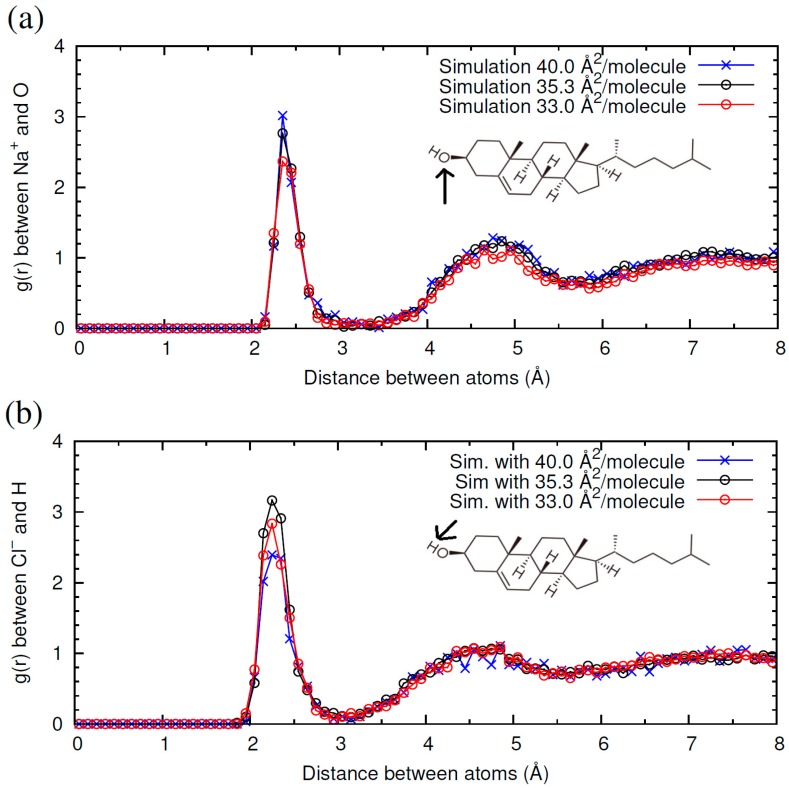
Radial distribution functions *g*(*r*) between NaCl electrolyte and CHOL headgroup obtained in three different MD simulations with different surface areas of CHOL monolayers: 33.1 Å^2^/molecule (red open circles); 35.3 Å^2^/molecule (black open circles) and; 40 Å^2^/molecule (blue crosses) (*T* = 298 K). (**a**) *g*(*r*) between Na^+^ of NaCl and oxygen atom in –OH headgroup of CHOL; (**b**) *g*(*r*) between Cl^−^ of NaCl and H atom in –OH headgroup of CHOL. The insets shows the molecular structure of CHOL as a reference. The relevant atom of the headgroup (H or O) is indicated with an arrow.

Another interesting consequence from the results shown in Figure 2 is that we do not observe significant adsorption or accumulation of ions at the water-CHOL interface (the residence time of ions near –OH groups is always on the order of a few picoseconds. However, the ions interact with the –OH groups located at the interface. This can be shown quantitatively by computing the radial distribution function *g*(*r*) between the ions and the relevant atom of the –OH headgroup of the CHOL molecule (oxygen for cations, hydrogen for anions). The strength of the interaction can be described by the first peak in this *g*(*r*) function. As shown in Figure 3a, the Na^+^ cation has the strongest interaction with CHOL for the largest area per molecule (40 Å^2^/molecule). As the area per molecule decreases, the height of this peak also decreases, revealing a weaker interaction. The behavior of Cl^−^ (Figure 3b) is opposite to that of Na^+^, being that the interaction of Cl^−^ with CHOL is stronger for the smallest surface area (35.3 Å^2^/molecule) and decreases as the surface area increases. This behavior can be understood by noting that, as the monolayer is more compressed (surface area decreases), the orientation of the –OH headgroup changes and the oxygen atom becomes less exposed to the aqueous phase, whereas the hydrogen atom becomes more exposed.

### 2.2. Comparison of Simulation Results for Different Electrolytes: NaI, NaCl, MgCl_2_ and CaCl_2_

From our simulation results in Section 2.1 for NaCl, it is clear that both cations and anions interact with CHOL. It is therefore interesting to compare how different combinations of anions and cations interact with CHOL. To this end, we have considered simulations at the same area per CHOL molecule (40 Å^2^/molecule) and same ionic strength but different electrolyte: NaCl, NaI, MgCl_2_ and CaCl_2_ (see Section 4 for technical details).

**Figure 4:**
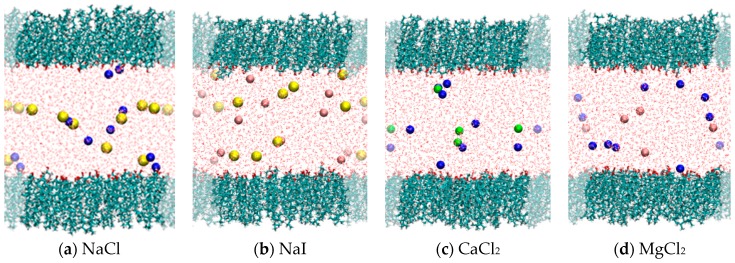
Snapshots from MD simulations (*T* = 298 K) corresponding to simulations at a fixed area per molecule (40 Å^2^/molecule) but different electrolyte. CHOL molecules are shown in bond representation; water is shown as lines and ions as spheres with Van der Waals radii. (**a**) NaCl (yellow spheres: Na^+^; blue spheres: Cl^−^); (**b**) NaI (yellow spheres: Na^+^; orange spheres: I^−^); (**c**) CaCl_2_ (green spheres: Ca^2+^; blue spheres Cl^−^) and; (**d**) MgCl_2_ (orange spheres: Mg^2+^; blue spheres: Cl^−^). The shaded region indicates periodic boundary conditions. This figure was made with VMD software [29].

Snapshots of the simulations for these four electrolytes are shown in Figure 4. We do not observe significant structural differences between the CHOL monolayers in all these cases at 40 Å^2^/molecule and different electrolytes. In all cases, the cholesterol monolayer forms the structure described in the previous subsection: a compact, planar structure with the –OH group in contact with the water phase and the hydrophobic core of the CHOL molecule oriented perpendicular to the interface. Thus, any difference observed in the interaction of ions with CHOL at 40 Å^2^/molecule has to be attributed to a specific interaction of the ions with the –OH group of CHOL rather than to a structural change in the monolayer. The analysis of *g*(*r*) functions obtained from simulations reveals such differences, as we discuss now in detail.

**Figure 5:**
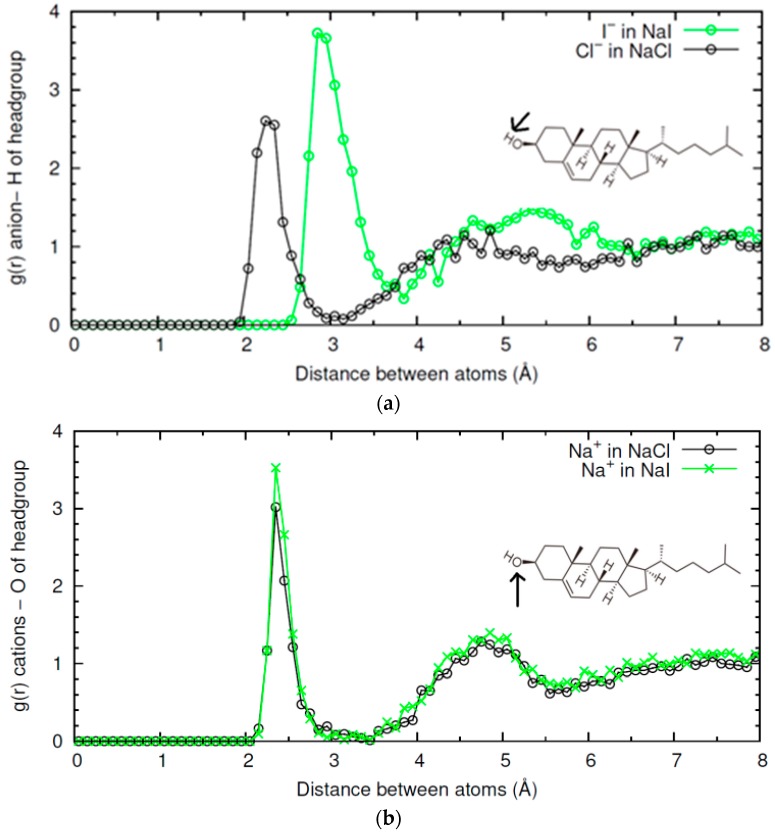
Radial distribution functions *g*(*r*) between NaCl or NaI ions and CHOL headgroup obtained in MD simulations (*T* = 298 K) (**a**) *g*(*r*) between Cl^−^ from NaCl (black open circles) or I^−^ from NaI (green open circles) and H atom in –OH headgroup of CHOL; (**b**) *g*(*r*) between Na^+^ from NaCl (black open circles) or NaI (green open circles), and oxygen atom in –OH headgroup of CHOL.

In Figure 5 we compare the *g*(*r*) functions characterizing the ion-CHOL interactions obtained for NaCl and NaI. As seen in Figure 5a, the first peak in the *g*(*r*) function is substantially higher for I^−^ than for Cl^−^. This implies that the interaction of the H atom of the –OH headgroup of CHOL with the poorly solvated I^−^ anion is stronger than with Cl^−^. The higher size of I^−^ also leads to a displacement of the position of the first peak in *g*(*r*). Interestingly, we see in Figure 5b that the interaction of Na^+^ with the –OH headgroup of CHOL is stronger in the case of NaI than in the case of NaCl. This result implies than the interaction of a cation with CHOL depends on the counterion.

**Figure 6:**
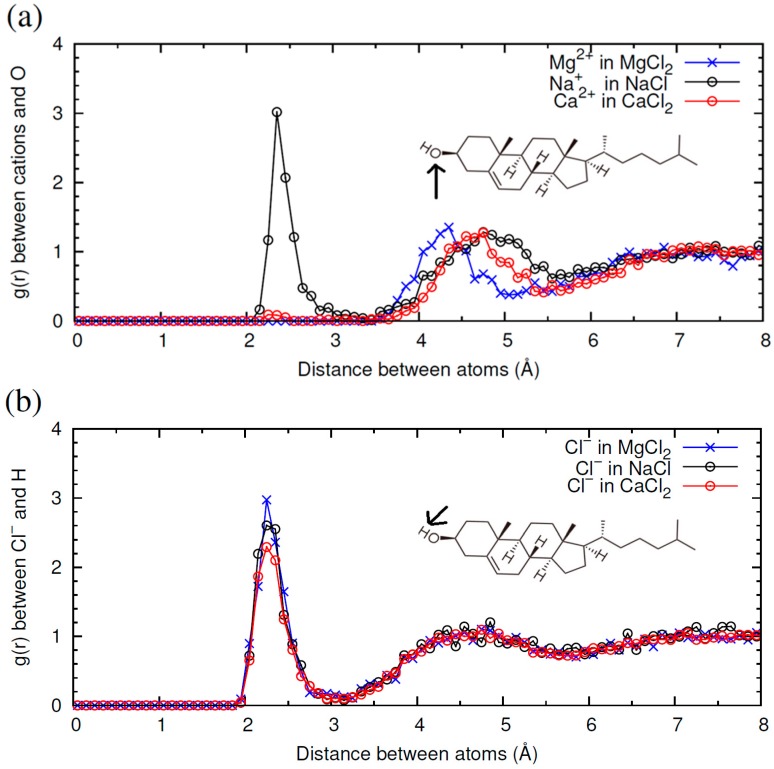
Radial distribution functions *g*(*r*) between ions from NaCl, CaCl_2_ or MgCl_2_ and CHOL headgroup obtained in three different MD simulations (*T* = 298 K). In the presence of NaCl (black open circles), CaCl_2_ (red open circles) or MgCl_2_ (blue crosses). (**a**) *g*(*r*) between different cations and oxygen atom in –OH headgroup of CHOL: Na^+^ from NaCl (black open circles), Ca^2+^ from CaCl_2_ (red open circles) or Mg^2+^ from MgCl_2_ (blue crosses); (**b**) *g*(*r*) between Cl^−^ from different electrolyte (NaCl, black open circles, CaCl_2_ red open circles, MgCl_2_ blue crosses) and H atom in –OH headgroup of CHOL.

Now we compare the results obtained with salts containing Cl^−^ and three different cations: Na^+^, Ca^2+^ and Mg^2+^. In Figure 6 we show the *g*(*r*) functions characterizing the ion-CHOL interactions. As shown in Figure 6a, in the case of Na^+^ -CHOL interaction, we have a first correlation peak with *g*(*r*) ≈ 3 at 2.5 Å, corresponding to contact between oxygen atoms and Na^+^ ions, and a small and broad secondary peak at 4.7 Å. This first peak of Na^+^ is absent in the case of Mg^2+^, but it is seen as a barely distinguishable peak in the case of Ca^2+^. For divalent cations, the first peak is located far from the oxygen atom of the CHOL –OH group, at *r* ≈ 4.3 Å for Mg^2+^ and at *r* ≈ 4.6 Å for Ca^2+^ (this small difference is due to the difference in size between the two cations). The height of this peak is also very low (*g*(*r*) ≈ 1.4), which indicates that the interaction of divalent cations with CHOL is substantially weaker than the interaction of Na^+^ with CHOL. By looking at the snapshots of the simulations, we see that divalent cations always approach the surface without losing their hydration shell. The obtained result can be interpreted by stating that Ca^2+^ and Mg^2+^ prefer to maintain their hydration shells instead of interacting with the headgroup of CHOL (more Mg^2+^ than Ca^2+^). This is in marked contrast with what happens in the interaction of these divalent cations with other phospholipids such as 1,2-Dipalmitoyl-sn-glycero-3-phosphoserine (DPPS), for example [4]. In the case of DPPS, divalent cations adsorb with large affinity. In the adsorbed state, the cations have a larger number of oxygen atoms (from phospholipids and water) in their first coordination shell than in bulk water. Mg^2+^ and Ca^2+^ also adsorb strongly onto liposomes of zwitterionic phospholipids, following a mechanism similar to that described by anionic liposomes [30]. Clearly, the behavior of CHOL differs from than reported to date for phospholipids. Due to its small headgroup with only one electronegative oxygen atom, CHOL has an unusually weak interaction with highly hydrated divalent cations. This interaction is essentially different from that observed between cations and other membrane lipids.

The interaction of each cation with the interface influences, in turn, the interaction of the Cl^−^ anion with CHOL. In Figure 6b, we show the radial distribution function between Cl^−^ and H atoms in the –OH headgroup of CHOL. In the three cases, we find the first peak of *g*(*r*) at the same location *r*, but the value of the peak follows the order MgCl_2_ > NaCl > CaCl_2_. This implies again that the interaction of a given ion (in this case Cl^−^) with CHOL depends on the counterion.

### 2.3. Experimental Results: Interaction of CHOL Monolayers With Subphases with Different Electrolytes: NaCl, NaI, MgCl_2_ and CaCl_2_

**Figure 7:**
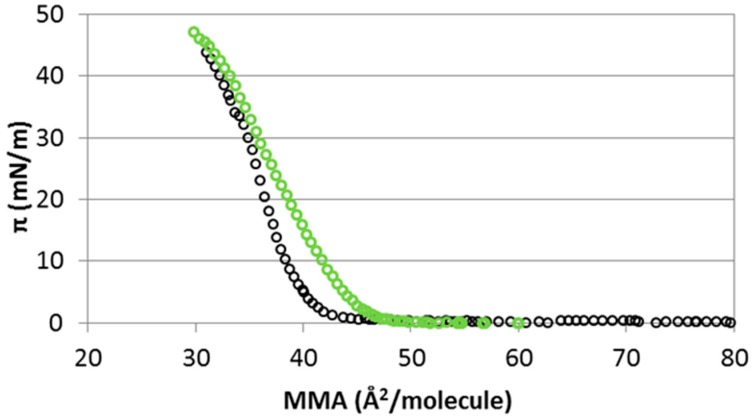
Isotherm of surface pressure (π) *vs.* area per molecule (MMA) of CHOL monolayers in a 15 mM subphase of monovalent salts (NaCl black open circles and NaI green open circles) (standard deviation <2%).

**Figure 8:**
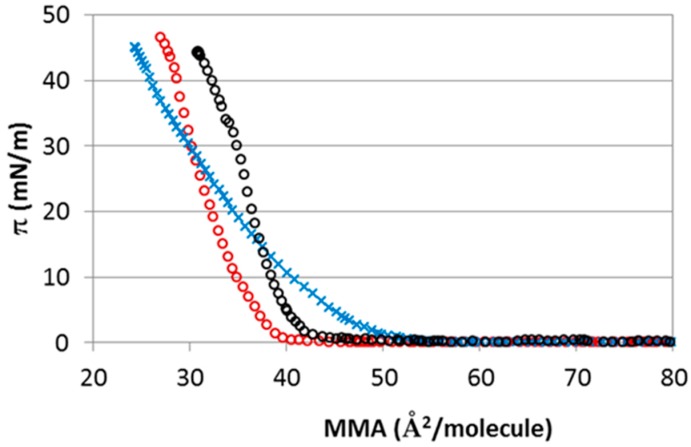
Isotherm of surface pressure (π) *vs.* area per molecule (MMA) of CHOL monolayers in a 5 mM subphase of MgCl_2_ (blue crosses), CaCl_2_ (red open circles) and in a 15 mM subphase of NaCl (black open circles) (standard deviation <2%).

As we have seen in the previous subsection, the simulation results show substantial ionic specific effects on CHOL monolayers. In order to address this question experimentally, we have measured the π-A isotherms of CHOL in the presence of the different electrolyte subphases. Figure 7 and Figure 8 show the effect of modifying the anion (Cl^−^ and I^−^) and cations (Ca^2+^ and Mg^2+^), respectively. It is important to recall here that a π-A isotherm covers a whole compression cycle of the monolayer while the simulations shown in Figure 5 and Figure 6 represent the situation at a single value of molecular area (40 Å^2^/molecule).

As expected from the simulations, we also experimentally obtain substantial specific ionic effects in the π-A isotherms. We will first discuss the results in Figure 7 and Table 1, corresponding to CHOL monolayers spread on NaCl and NaI subphases. Clearly, the nature of the anion (Cl^−^ or I^−^) induces various changes in the isotherm as both the shape and the location of the characteristic points of the isotherm are affected by the nature of the anion. The isotherm of CHOL obtained in the presence of I^−^ lifts off at higher molecular areas. This is consistent with the simulation results reported in Figure 5 at a molecular area of 40 Å^2^/molecule, which reveal a stronger interaction between CHOL/I^−^ than between CHOL/Na^+^. The shape of the isotherm is also different for I^−^ and Cl^−^ subphases. This different shape is reflected in the different Gibbs elasticity values shown in Table 1. Gibbs elasticity reflects the slope of the monolayer of CHOL, which is less pronounced in the presence of I^−^. Accordingly, the obtained cohesivity of the surface layer is smaller, meaning that the state of the monolayer is somehow less condensed in the presence of I^−^. Finally, collapse pressure increases slightly in the presence of I^−^ in the subphase, as compared with Cl^−^ (Table 1).

Consider now the interaction of CHOL with subphases containing different cations (Mg^2+^, Ca^2+^) but maintaining the same counterion (Cl^−^). In order to maintain the same ionic strength as in the case of monovalent ions discussed before, the concentrations considered here are 5 mM. Figure 8 shows the isotherms of CHOL monolayer spread on CaCl_2_ and MgCl_2_ subphase (the isotherm recorded on NaCl subphase has been included for comparison). The π-A isotherms obtained in the presence of Ca^2+^ and Na^+^ display similar shape, but the isotherm is displaced to lower molecular areas for Ca^2+^, thus corresponding to a lower lift-off area (Table 1). The shape and slope of CHOL isotherms at NaCl and CaCl_2_ subphase are similar, and this is reflected in their similar Gibbs elasticity values (see Table 1). Conversely, the presence of Mg^2+^ induces profound changes in the shape of the monolayer as compared to Ca^2+^ and Na^+^. The slope of the monolayer of CHOL in the presence of Mg^2+^ is less pronounced. This is reflected in the lower Gibbs elasticity of CHOL in the presence of Mg^2+^ as seen in Table 1. Lower elasticity values are usually interpreted as an indication of smaller molecular cohesivity within the surface layer. Comparing the isotherms for the three subphases, we note that at the same area per CHOL of 40 Å^2^/molecule, the obtained π follows the sequence Mg^2+^ > Na^+^ > Ca^2+^, which coincides with the sequence of the first peak in the *g*(*r*) functions obtained by simulations shown in the bottom panel of Figure 6.

Another magnitude of interest of the isotherm is the area per molecule obtained at the highest surface pressures, which is a measure of the condensing effect of the ions. These areas follow the sequence Mg^2+^ > Ca^2+^ > Na^+^, indicating that the condensation effect induced in the monolayer is more pronounced for divalent cations. Moreover, collapse pressures are similar in all cases (see Table 1), being just slightly higher for divalent cations consistent with a higher condensation effect induced for divalent cations.

## 3. Discussion

Our study, which combines Langmuir Film Balance experiments and MD simulations, clearly demonstrates the presence of specific ionic effects in CHOL monolayers for the electrolytes considered here (NaI, NaCl, CaCl_2_ and MgCl_2_).

The shape of the experimental π-A isotherms are qualitatively similar for NaCl and CaCl_2_, but differ substantially from the shapes observed for the other considered electrolytes (MgCl_2_ and NaI, see Figure 7 and Figure 8). The fact that the isotherms display both quantitative and qualitative differences for each electrolyte makes it difficult to propose a series or ranking of the effect of the ions, ranging from those having more impact to less impact on the isotherms, contrary to what is usually done in specific ionic effects (we recall here the so-called Hoffmeister series, popular in colloidal science [2]). This is clearly seen looking at the characteristics of the π-A isotherms, compiled in Table 1. On the one hand, the Gibbs elasticity decreases with the series NaCl > CaCl_2_ > NaI > MgCl_2_. On the other hand, the lift-off molecular area A_0_ (Table 1) increases with the series CaCl_2_ < NaCl < NaI ≈ MgCl_2_. These series do not seem to correlate with any obvious property of the ions, such as size (I^−^ is the bigger one and Mg^2+^ is the smallest), charge, polarizability or hydration.

Our atomistic modelling (MD simulations) also shows a complex picture emerging at the microscopic scale. First, our results show that the ions do not adsorb at the interface, but interact with the –OH group of the CHOL molecules. Our results suggest that less hydrated ions have stronger interactions with the –OH headgroup of cholesterol than more hydrated ions, a picture consistent with the fact that the cholesterol molecule is only weakly polar and is mostly hydrophobic. For example, I^−^ interacts stronger with cholesterol than Cl^−^ (as measured by the radial distribution function), and Na^+^ also interacts more strongly with cholesterol than Ca^2+^ or Mg^2+^. However, our results show that the interaction of a particular ion with the cholesterol film strongly depends on its counterion. For example, the interaction of Na^+^ with the –OH headgroup of cholesterol is stronger with I^−^ as counterion (as compared with the results obtained for Na^+^ in presence of Cl^−^). Also, the interaction of Cl^−^ with the –OH headgroup of cholesterol follows the sequence CaCl_2_ < NaCl < MgCl_2_. The existence of a counterion effect was suggested in some previous theoretical works (see, for example, [31]) as a general phenomenon in biological systems as a consequence of ionic polarization. However, polarization cannot be the driving force here for the counterion effect as it is absent in our simulations, so its origin here remains unknown.

In summary, our results show that CHOL monolayers have an interesting and atypical specific behavior depending on the ionic subphase, which challenges our current theoretical understanding of specific ionic effects. Well-known concepts such as ionic series seem to be of little use in this case, and new effects such as the counterion effect need to be taken into account. Whether these features are specific to CHOL or can perhaps be found with films of other biological molecules is an interesting question that deserves to be considered in detail in the near future.

## 4. Materials and Methods

### 4.1. Materials

CHOL was purchased from Sigma (≥99% purity, C8667) and used as received. Spreading solutions of CHOL were prepared in chloroform/methanol 4:1 (*v*/*v*) mixture (HPLC grade, ≥99%, Aldrich). CaCl_2_ solution (Fluka, 21114), MgCl_2_ solution (Fluka 65020), NaCl (Sharlau S02241000), NaI (Sharlau, SO08350250) were all used as received.

Ultrapure water, cleaned using a Milli-Q water purification system (0.054 uS, Merck KGaA, Darmstadt, Germany), was used for the preparation of buffer solutions. All glassware was washed with 10% Micro-90 cleaning solution and exhaustively rinsed with tap water, isopropanol, deionized water and ultrapure water in this sequence. All other chemical used were of analytical grades and used as received.

### 4.2. Experimental

CHOL monolayers at the air-water interface were formed in a Langmuir Film Balance equipped with a paper Wilhelmy plate surface pressure measuring system (KSV), as in previous works with cholesterol monolayers [26]. Although this is a standard experimental technique, the interpretation of the Wilhelmy plate measurement has to be done carefully in the case of rigid monolayers. A small increase in pressure at the periphery leads to a weaker increase at the plate, the weakening being governed by the ratio of shear to bulk modulus [32,33]. In the case of rigid monolayers, the surface pressure should be interpreted while considering that parts of the monolayer away from the plate may be under a compressive stress even if a positive surface tension is measured at the plate.

The trough was thoroughly cleaned before every measurement with the following sequence: 10% Micro-90^®^ cleaning solution; tap water; isopropanol; distilled water and; ultrapure water. The trough was filled with the adequate subphase (15 mM NaCl, 15 mM NaI, 5 mM CaCl_2_ or 5 mM MgCl_2_), in order to maintain a constant ionic strength (*I* = 15 mM). We selected these electrolyte concentrations based on previous studies of lipid monolayers containing cholesterol [22,24]. The absence of surface active impurities was tested within the whole compression range before every experiment obtaining values of surface pressure π < 0.2 mN/m. After the equilibration of the subphase, 50 µL of CHOL solution (0.227 mg/mL) in chloroform: Methanol 4:1 were carefully spread on the subphase using a microsyringe (Hamilton^®^, St. Louis, MO, USA). After 20 min to ensure the evaporation of solvent, the surface pressure-area (π-A) isotherm was recorded at a constant rate of 5 mm/min. The reproducibility of the π-A isotherm of CHOL monolayer spread on different subphases was tested by at least three different measurements, carried out in triplicate for independent samples obtaining standard deviation <2%. Final values are expressed as mean values of replicates ± standard deviations according to statistical analysis tools. The Langmuir film balance lies on an anti-vibration table and is covered with a transparent Plexiglas case in order to avoid perturbation of the air-water interface by air stream and/or dust deposition. The temperature of the subphase was controlled by a circulation water system thermostat at 25.0 ± 0.1 °C.

The surface Gibbs elasticity of the monolayer was calculated directly from the π-A isotherm using

(1) $$\varepsilon_{0}=-A{\left(\frac{d\pi }{dA}\right)}_{T}$$

The lift-off area (*A*_0_) corresponds with the value of area per molecule at which the surface pressure begins to increase; hence, where the isotherm lifts off. The collapse pressure is the surface pressure at the highest compression state of the monolayer before collapse.

### 4.3. Methods for MD Simulations

Molecular dynamics (MD) simulations are based on the numerical solution of the Newtonian equations of motion for all atoms of a molecular system constrained to the given thermodynamic conditions. All MD simulations reported here were performed employing the NAMD software (version 2.9) [34]. The interactions between molecules were modelled employing the CHARMM force field, including a new parameterization for the CHOL molecule [35,36]. The employed model for the water molecule was TIP3P, as is standard in CHARMM. The I^−^ ion has no standard CHARMM parameters, so we modelled it using the parameters employed in previous studies of ionic specific effects [37].

The systems considered in our simulations consist of a water film in vacuum (air is not simulated) covered with a CHOL monolayer at each water interface, as seen in Figure 2. We considered different values of the area per molecule of the CHOL monolayer and different subphases. In all the simulations we had a total of 100 CHOL molecules (50 in each layer) and 2470 water molecules. In the simulations with NaCl or NaI we had 8 cations and 8 anions. In the simulations with divalent ions we had 4 divalent cations and 8 anions.

The Newtonian equations of motion for all the molecules were solved with a 2 fs time step. Electrostatic interactions were computed using the particle mesh Ewald summation method (PME), with the standard settings in NAMD (1 Å spatial resolution and were updated every two time steps). Lennard-Jones interactions were truncated at 1.2 nm, employing a switching function starting at 1.0 nm. The temperature was kept constant at 25 °C using the Langevin thermostat with a relaxation constant of 1 ps^−1^.

The initial configuration of the system (CHOL monolayers, water slab and NaCl subphase) was generated by using the membrane builder [38,39] from the CHARMM-GUI [40] web-based interface. The optimized simulation box had equilibrium lateral dimension of *L*_x_ = *L*_y_ = 6.32 Å, which corresponds to 40 Å^2^/molecule. From this configuration, we generated initial configurations for all other considered electrolyte (NaI, MgCl_2_, CaCl_2_) by replacing Na^+^ or Cl^−^ by the required ion and performing an additional energy minimization after the ion replacement.

Production runs of the molecular dynamics simulations with a surface area of 40 Å^2^/molecule and different electrolytes (NaCl, NaI, MgCl_2_, CaCl_2_) were performed in the constant number of particles (N), volume (V) and temperature (T) (NVT) ensemble, with a total of 80 ns of simulations (20 ns for each electrolyte).

In the case of NaCl, the final configuration after the NVT production runs was employed to generate initial configurations for systems with smaller areas per CHOL molecule. To this end, we performed short (1 ns) simulation runs with an applied external force in the XY plane acting over all atoms, using the TCL force functionality of NAMD. Using two different values of the forces, we obtained monolayers with *L*_x_ = *L*_y_ = 42.0 Å and *L*_x_ = *L*_y_ = 40.7 Å, corresponding to areas per CHOL molecule of 35.3 Å^2^/molecule and 33.1 Å^2^/molecule, respectively. These configurations were energy minimized, thermally equilibrated with a short NVT run (to ensure the stability of the total energy and the equipartition temperature) and, finally, data was collected from 20 ns NVT production runs for each area per molecule.

All snapshots and *g*(*r*) functions were obtained from the production runs using VMD (version 1.9) [29].

## Figures and Tables

**Table 1:** Gibbs elasticity values at the maximum, lift-off molecular area ***A*_0_** and collapse pressure *Π*_coll_ for CHOL monolayer at different subphases.

Salts	Elasticity (mN/m)	*A*_0_ (Å^2^/molecule)	*Π*_coll_ (mN/m)
NaCl (15 mM)	245 ± 5	40 ± 2	44 ± 2
NaI (15 mM)	160 ± 5	45 ± 2	46 ± 2
CaCl_2_ (5 mM)	230 ± 4	38 ± 2	46.0 ± 1.0
MgCl_2_ (5 mM)	140 ± 4	46 ± 2	46.2 ± 1.5

## Abbreviations

The following abbreviations are used in this manuscript:

CHOL	Cholesterol molecule
DPPS	1,2-Dipalmitoyl-sn-glycero-3-phosphoserine
MD	Molecular Dynamics Simulations
MMA	area per molecule
NVT	Molecular dynamics simulations performed at constant number of particles (*N*), volume (*V*) and temperature (*T*)
π	Surface pressure
